# Heterogeneous mutation pattern in tumor tissue and circulating tumor DNA warrants parallel NGS panel testing

**DOI:** 10.1186/s12943-018-0875-0

**Published:** 2018-08-28

**Authors:** Qiaomei Guo, Junlei Wang, Jianfeng Xiao, Lin Wang, Xiaomeng Hu, Wenjun Yu, Gang Song, Jiatao Lou, JianFeng Chen

**Affiliations:** 1 Department of Laboratory Medicine, Shanghai Chest Hospital, Shanghai Jiao Tong University, Shanghai, China; 2 State Key Laboratory of Cell Biology, CAS Center for Excellence in Molecular Cell Science, Shanghai Institute of Biochemistry and Cell Biology, Chinese Academy of Sciences, University of Chinese Academy of Sciences, 320 Yueyang Road, Shanghai, 200031 China; 3 Department of Research and Development, Shanghai Zhengu Biotech Ltd., Shanghai, China

**Keywords:** Liquid biopsy, Circulating tumor DNA, NGS,non-small-cell lung cancer, Somatic mutations, Concordance

## Abstract

Liquid biopsy by genotyping circulating tumor DNA (ctDNA) has provided a non-invasive approach in assessing tumor genomic alterations in clinical oncology. However, emerging evidence in clinical settings has shown significant discordance in the genomic alterations between matched tumor tissue and blood ctDNA samples, and even between the same set of blood samples analyzed on different testing platforms. Thus, it is necessary to study underlying causes of discrepancies in these studies by genotyping tumor tissue and ctDNA in parallel using next generation sequencing (NGS) panels based on the same technology. Here we enrolled 56 non-small-cell lung cancer (NSCLC) patients and evaluated tumor tissue genotyping and ctDNA based liquid biopsy by parallel NGS panel testing and compared different sample preparation conditions. Somatic mutations in plasma cell-free DNA (cfDNA) were detected in 63.6% patients with early-stage NSCLC and 60% patients with advanced-stage NSCLC. The overall concordance between matched formalin-fixed paraffin-embedded sample and cfDNA was 54.6% in early-stage NSCLC patients and 80% in advanced-stage NSCLC patients. The positive concordance rate was 44.4% and 71.4% in early-stage and advanced-stage patients, respectively. Using fresh frozen tumor samples did not improve the overall concordance rate between matched tumor tissue and cfDNA. Processing blood samples beyond 4 h after blood draw significantly decreased the detection rate of somatic mutations in cfDNA. Thus, the concordance rate between tumor tissue-based and ctDNA-based genotyping in clinical samples can be affected by multiple pre-analytical, analytical and biologic factors. Parallel NGS panel testing on both sample types for each patient may be warranted for effective guidance of cancer targeted therapies and possible early detection of cancer.

**Electronic supplementary material:**

The online version of this article (10.1186/s12943-018-0875-0) contains supplementary material, which is available to authorized users.

## Main text

Genotyping tumor tissue biopsy has become a standard practice in clinical oncology for cancer patient management. Recently, liquid biopsy using circulating tumor DNA (ctDNA) has provided a non-invasive approach in assessing tumor genomic alterations for cancer early detection, personalized therapy and treatment monitoring [1–3]. Commercially available tissue genotyping and liquid biopsy tests, including FoundationOne (F1), Guardant360 (G360) and PlasmaSELECT (PS), self-reported high accuracy, sensitivity and specificity to detect tumor-specific genomic alterations [4–6]. However, independent studies reported significant discordance in testing results between matched tumor tissues and ctDNA generated on F1 and G360 [7]. One recent study reports high discordance in ctDNA results for the same set of blood samples between G360 and PS [8]. The inaccurate genetic profiling in actual clinical settings has raised serious concerns about the risks of misguiding treatment decisions to cancer patients. In these studies, tissue and blood specimens were shipped to different vendors for DNA extraction, library preparations, targeted NGS and data analysis. The underlying causes of discrepancies in these studies are difficult to be identified as the testing results were generated on clinical specimens across different testing platforms. Thus, genotyping tumor tissue and ctDNA in parallel on the same testing platform is important for clarifying this question prior to rigorous cross-platform comparisons using a large number of clinical samples.

**Fig. 1:**
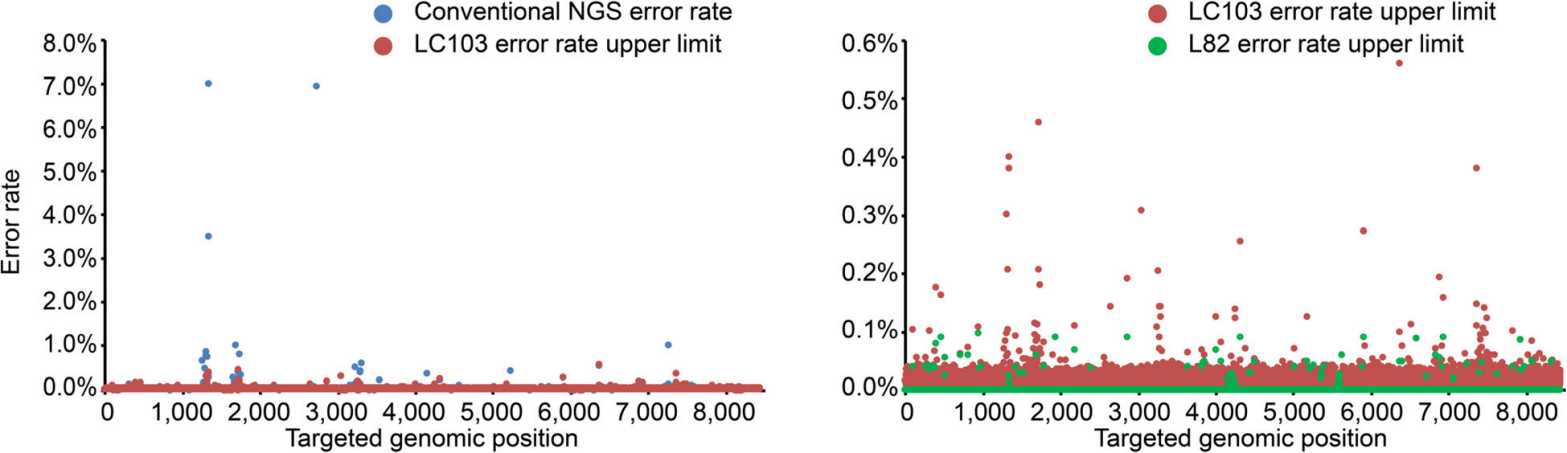
Error rate reduction in LC103 and L82 gene panels compared to conventional NGS for Q30 bases. LC103 targets 103 regions of interest in 22 lung and colon cancer related genes**.** L82 interrogates 82 regions in 17 overlapping genes with LC103. Data were generated on Illumina NextSeq. Only the overlapping bases between two panels are plotted. At each base position, error rates are calculated by dividing the number of error alterations by the total base coverage using the data from cell line FFPE references after removing known mutations, and from healthy individuals analyzed for cfDNA analysis. The error rate of LC103 is well below 1%, which allows reliable mutation detection above mutant allele frequency (MAF) of 2% in tumor tissue. For all mutations in the Multiplex Reference standards (FFPE DNA or sections, Horizon Discovery), the observed allele frequencies are consistent with the expected allele frequencies. In the L82 dataset (green), recurrent background errors (< 0.1% of error rate) are shown in the figure. These errors appear at non-hotspot positions and can be further reduced using a position-specific unbiased approach

To evaluate tumor tissue genotyping and ctDNA-based liquid biopsy by parallel NGS panel testing, we enrolled a total of 56 newly diagnosed early-stage (stages I and II) and advanced-stage (stages III and IV) non-small-cell lung cancer (NSCLC) patients (Table 1, and Additional file 1: Table S1). Blood samples from these patients were collected within 0–26 days before surgery. Each patient had matched formalin-fixed paraffin-embedded (FFPE) tumor tissue and germline DNA extracted from white blood cells. Matched fresh frozen (FF) tissue was also available for 21 out of 56 patients. We analyzed genomic alterations in cfDNA, matched germline, FFPE and FF DNA samples using NGS targeted sequencing panels. The Lung and Colon Cancer Panel (LC103) and the high sensitivity Lung Cancer Panel (L82) from Pillar Biosciences Inc. (Fig. 1 and Additional file 2) were used for tumor tissue and cfDNA samples, respectively. Different plasma sample processing time is also compared.

**Table 1:** Clinical characteristics of NSCLC patients with both tissue and ctDNA NGS testing

Characteristic	Number	Percentage(%)
Age, years		
Mean (SD)	59.73	
Median (range)	60 (42–82)	
Gender		
Female	29	51.79
Male	27	48.21
Stage		
I	38	67.86
II	7	12.50
III	9	16.07
IV	2	3.57
Cytological diagnosis		
Adenocarcinoma	46	82.14
Squamous cell carcinoma	10	17.86

**Fig. 2:**
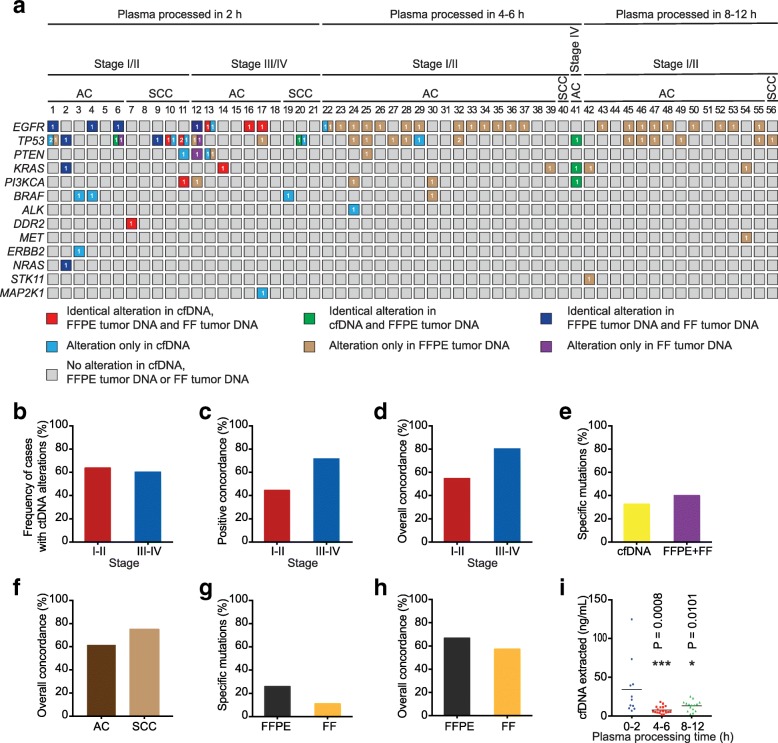
Mutation analysis of matched tumor tissue and cfDNA from NSCLC patients by parallel NGS panel testing**. a** Mutational landscape of matched cfDNA and FFPE, FF tissue DNA from NSCLC patients. Each column represents 1 patient. Only alterations in overlapping base positions of L103 and L82 gene panels were included. For Patients 1–21, matched cfDNA, FFPE and FF tumor tissue DNA were sequenced. For Patients 22–56, matched cfDNA and FFPE tumor tissue DNA were sequenced. Number in the square indicates the number of different mutations in each gene. **b-h** Whole blood was collected from NSCLC patients1–21 in EDTA tubes and processed within 2 h post venesection. Frequency of cases with detectable mutations in cfDNA in early-stage NSCLC (stages I and II) and advanced-stage (stages III and IV) NSCLC (**b**), positive concordance rate for genomic alterations in plasma and FFPE tumor tissue (**c**), overall concordance rate for genomic alterations in plasma and FFPE tumor tissue (**d**), frequency of specific mutations in tumor tissue biopsies (including both FFPE and FF) and plasma cfDNA samples (**e**), overall concordance of genomic alterations between plasma and FFPE tumor tissue in adenocarcinoma (AC) and squamous cell carcinoma (SCC) (**f**), specific variants in matched FFPE and FF biopsies (**g**) and overall concordance of genomic alterations between matched tumor tissue sample (FFPE or FF) and cfDNA (**h**). **i** Whole blood was collected from three groups of early stage NSCLC patients (patients 1–11, 22–40 and 42–56) in EDTA tubes, stored at RT and processed within 2 h, 4–6 h and 8–12 h post venesection, respectively. After cfDNA was isolated from plasma, the cfDNA concentration was determined by Qubit 2.0. Means for each group are represented by the black bars in the columns analyzed. The concentrations of cfDNA in plasma samples processed within 2 h, 4–6 h and 8–12 h are 34.27 ng/mL (95% CI, 10.12–58.43), 7.64 ng/mL (95% CI, 5.43–9.85) and 13.02 ng/mL (95% CI, 9.3–16.73), respectively

We examined somatic alterations in 21 matched NSCLC tumor biopsies and plasma cfDNA samples, which were obtained at the time of surgery and processed within 2 h after blood collection. Somatic mutations in cfDNA were detected in 7 out of 11 (63.6%) early-stage NSCLC patients and 6 out of 10 (60%) patients with advanced-stage NSCLC (Fig. 2a, b and Additional file 3: Table S2). In 14 low frequency cfDNA-specific alterations detected by L82 panel sequencing, 13 of them were also detected in cfDNA by droplet digital PCR (ddPCR) (Additional file 4: Table S3). No somatic variant was detectable in 5 patients (2 stage I and 3 stage III).

We further compared the concordance between FFPE tumor biopsy and ctDNA genomic profiling. The positive concordance rate was 44.4% (4/9) and 71.4% (5/7) in early-stage and advanced-stage NSCLC patients, respectively (Fig. 2a and c). Three patients (No.7, 14, 16) showed complete concordant genomic alterations in tumor tissue and cfDNA. Five patients (No.5, 8, 15, 18, 21) displayed negative concordance with genomic alterations detected in neither tumor tissue nor cfDNA. The overall concordance was 54.6% (6/11) in early-stage patients and 80% (8/10) in advanced-stage patients (Fig. 2a and d), consistent with recent findings [1]. Nevertheless, the discordance rate at the mutation level was high, similar to the reported results [7–9]. Only 22.5% (9/40) of somatic alterations were detected in all three sample types (FF, FFPE and cfDNA) and 2 additional concordant alterations were identified in both FFPE and cfDNA (Fig. 2a). In contrast, 32.5% (13/40) and 40% (16/40) mutations were cfDNA-specific and tissue-specific, respectively (Fig. 2a and e). Four high frequency mutations (15–51.03%) in FFPE and FF tissues were not detected in cfDNA by both L82 panel sequencing and ddPCR (Additional file 4: Table S3). One high frequency mutation (TP53 c.481G > A) in FFPE and FF tissues were only detected in cfDNA by ddPCR but not by L82 panel sequencing, presumably because the ctDNA copies corresponding to the variant was extremely low in cfDNA. In addition, the overall concordance was 61.5% (2/6 in early-stage and 6/7 in advanced-stage) in lung adenocarcinoma patients and 75% (4/5 in early-stage and 2/3 in advanced-stage) in squamous lung carcinoma patients (Fig. 2f). Collectively, genotyping FFPE tumor tissue and ctDNA in parallel on the same testing platform showed significant discordance in genomic alterations in tumor tissue and cfDNA, suggesting that intrinsic biological mechanisms might affect the concordance between tumor biopsy and ctDNA genomic profiling in different patients.

Previous studies have indicated that DNA damage in FFPE is a major source of erroneous identification of somatic variants with low to moderate (1 to 5%) allelic frequencies [10]. This error has also been attributed to the discrepancies between tissue biopsy and ctDNA. Therefore, we collected FF tissue in a different region of each tumor from 21 patients and performed genotyping analyses on matched FFPE and FF samples. In a total of 27 tissue somatic variants, 63% (17/27) are concordant between matched FF and FFPE samples. 25.9% (7/27) and 11.1% (3/27) of somatic mutations are FFPE- and FF-specfic, respectively (Fig. 2a, g, and Additional file 5: Table S4). The overall concordance rate between matched FFPE sample/cfDNA and FF sample/cfDNA were 66.7% (14/21) and 57.1% (12/21), respectively, (Fig. 2a and h). These results suggest that DNA damage in FFPE is not the major factor contributing to the discordance between tumor tissues and ctDNA in this study.

We next evaluated the impact of blood sample processing on mutation detection in cfDNA. Whole blood was collected from three groups of NSCLC patients in EDTA tubes by a single operator following the same protocol and then processed within 2 h, 4–6 h and 8–12 h post venesection. We found that cfDNA concentration in plasma processed within 2 h was significantly higher than that within 4–6 h or 8–12 h (Fig. 2i). The low cfDNA concentration at 4–6 h is most likely caused by cfDNA degradation, whereas the elevated cfDNA concentration at 8–12 h might be due to the genomic DNA released from leukocytes. The positive detection rate decreased significantly with the extended processing time (Fig. 2a). All 5 concordant cases had no genomic alterations detected in both tumor and plasma. Our results highlight the importance of processing blood samples in the EDTA tubes within 2 h to improve mutation detection sensitivity in cfDNA.

In summary, significant discordance in the genomic alterations of the matched tumor tissues and ctDNA was observed by genotyping tumor tissue and ctDNA in parallel using the same NGS panel platform. Many pre-analytical, analytical and biologic factors may affect the genotyping results on tissue and ctDNA in a clinical oncology setting. Among these factors, the intrinsic heterogeneous mutation pattern in tumor tissues and ctDNA can jeopardize the clinical benefit of precision medicine. Further understanding biologic factors that affect ctDNA release are needed. Improving the assay sensitivity and specificity of either genotyping approach alone is unlikely to resolve this discordant genotyping issue. To enhance assay accuracy and clinical utility, parallel NGS panel testing on multiple sample types for each patient may be warranted for effective guidance of cancer targeted therapies and possible early detection of cancer. International standards for tumor molecular profiling using tumor tissues and ctDNA should be established.

## Additional files

Additional file 1: **Table S1.** Clinical characteristics of enrolled NSCLC patients. (XLSX 12 kb)

Additional file 2: Material and methods. (DOCX 29 kb)

Additional file 3: **Table S2.** Somatic alterations detected in cfDNA of NSCLC patients. (XLSX 13 kb)

Additional file 4: **Table S3.** Validation of ctDNA variants by ddPCR. (XLSX 10 kb)

Additional file 5: **Table S4.** Somatic alterations detected in tumor of NSCLC patients. (XLSX 15 kb)

## Abbreviations

cfDNA: Cell-free DNA
ctDNA: Circulating tumor DNA
ddPCR: Droplet digital PCR
FF: Fresh frozen
FFPE: Formalin-fixed paraffin-embedded
MAF: Mutant allele frequency
NGS: Next-generation sequencing
NSCLC: Non-small-cell lung cancer
