# Supplementary material for: Heterogeneous mutation pattern in tumor tissue and circulating tumor DNA warrants parallel NGS panel testing
Source: Mol Cancer. 2018 Aug 28;17:131. doi: 10.1186/s12943-018-0875-0 (PMC6114875; doi:10.1186/s12943-018-0875-0)
Supplement: Supplementary file 2 — Material and methods. (DOCX 29 kb) [file 12943_2018_875_MOESM2_ESM.docx]

**Materials and methods**

**Patient enrollment**

This study was approved by the Institutional Review Board of Shanghai Chest Hospital. NSCLC patients who were newly diagnosed without surgery and any treatment were enrolled. Informed consent was obtained from all subjects for research in this study. Histological subtype, tumor size, and the presence of pleural and lympho-vascular invasion were evaluated by at least two pathologists. All participants were not accompanied with any other malignant.

**Sample collection and processing**

Peripheral blood was collected in EDTA vacutainer tubes (BD Biosciences, Franklin Lakes, New Jersey, USA). Blood samples were processed within the specified time (0-2 h, 4-6 h, 8-12 h) after collection. Plasma was separated by centrifugation at 1900g, 4 °C for 10 min. Then the plasma was centrifuged at 16,000g and 4 °C for 10 min to remove additional cellular debris. The cell pellet from the initial spin was used for isolation of germline genomic DNA from PBLs. The supernatant and PBLs were stored at -80 °C. Matched tumor tissues were macro-dissected under a dissecting microscope to ensure a neoplastic cellularity of >60%. Matched fresh tumor tissues were frozen at -80 °C until use. Matched formalin-fixed, paraffin-embedded (FFPE) tumor specimens were obtained according to the standard histopathologic procedures.

**DNA purification and quantification**

Circulating DNA was isolated from 1.5-5.4 mL plasma with the QIAamp Circulating Nucleic Acid Kit (Qiagen, Hilden, Germany) following the manufacture’s instruction. Germline DNA and fresh frozen tumor DNA were extracted using the QIAamp DNA Mini Kit (Qiagen). Matched FFPE tumor DNA was purified using GeneRead DNA FFPE Kit (Qiagen). DNA samples were quantified by Qubit 2.0 Fluorometer (Thermo Fisher Scientific, Waltham, MA, USA). Quality of cfDNA was analyzed using Bioanalyzer 2100(Agilent Technologies, Santa Clara, CA, USA).

**Next-generation sequencing library construction and Sequencing**

For each patient, 20 ng germline DNA and tumor tissue DNA and 10-50 ng cfDNA were used for library construction, respectively. Indexed Illumina NGS Libraries were prepared and assessed as previously described [1], except using new adapter designs for molecular barcoding. In short, a linear PCR is performed for 16 cycles to attach the UIDs to the original DNA. Subsequently, after removing the unused UID-primers, the UID-tagged molecules are then exponentially amplified with the other gene-specific primer and two universal primers. The quality of libraries was assessed on the Bioanalyzer 2100. Then the libraries were quantified by Qubit 2.0 fluorometer and subjected to 150 bp paired-end runs on Illumina NextSeq500 to achieve more than 200x, 2000x, and 20,000× coverage for germline, tumor DNA and cfDNA libraries, respectively.

The Lung and Colon Cancer Panel (LC103) and the high sensitivity Lung Cancer Panel (L82) were from Pillar Biosciences Inc. LC103 targets 103 regions of interest in 22 lung and colon cancer related genes**.** In an independent validation study using residual clinical specimens, the LC103 panel demonstrated a good concordance with the Ion Torrent Cancer Hotspot Panel v2 (CHPv2; Life Technology) and a high degree of reproducibility in variant calls at both average and extremely low FFPE DNA inputs [2]. L82 interrogates 82 regions in 17 overlapping genes with LC103. Through a combination of high fidelity Taq polymerase, unique identifiers, redundant pair-end sequencing, the error correction algorithm and removal of matched germline mutations, the error rate of L82 was significantly reduced to reproducibly detect mutations at cutoff values of 0.1% and 0.2% MAF in hot spots and other positions, respectively.

**Variant Calling**

PiVAT^TM^ (Pillar Biosciences Inc.) was used for data analysis. In addition to the bioinformatics workflow described previously [1], PCR errors and sequencing errors are reduced to be well below 1% VAF through the PiVAT error correction algorithm for the detection of somatic mutations in tumors. A UID caller in the PiVAT^TM^ was used to detect somatic mutations in the ctDNA.

**Droplet dPCR analysis of plasma ctDNA**

All custom ordered primer and probe pairs were designed by Life Technologies. The ddPCR analysis was performed on a QX200 ddPCR system (Bio-Rad, Hercules, CA, USA). Results were analysed using QuantaSoft (version 1.6.6) software [3].

**Statistical Analysis**

In this study, Positive concordance was defined at the patient level as detecting an identical sequencing mutation in matched tumor tissues and ctDNA. Overall concordance was defined at the patient level as detecting an identical sequencing mutation or not detecting an alteration in matched tumor tissues and ctDNA. One-way ANOVA was used to compare the concentrations of extracted cfDNAs with different plasma processing time. All statistical analyses were performed using SPSS Statistics version 24 (IBM Corp, Chicago, IL, USA) and a P-value less than 0.05 was deemed statistically significant.

Reference

1. Schenk D, Song G, Ke Y, Wang Z. Amplification of overlapping DNA amplicons in a single-tube multiplex PCR for targeted next-generation sequencing of BRCA1 and BRCA2. *PLoS One* 2017, 12:e0181062.

2. Peterson J, Blumental dAF, Wang Z, Wells W, Tsongalis G. Evaluaiton of the Pillar NGS SLIMamp Lung and Colon Hot Spots Panel. *J Mol Diagn* 2017, 18:1017.

3. Hindson BJ, Ness KD, Masquelier DA, Belgrader P, Heredia NJ, Makarewicz AJ, Bright IJ, Lucero MY, Hiddessen AL, Legler TC, et al. High-throughput droplet digital PCR system for absolute quantitation of DNA copy number. *Anal Chem* 2011, 83:8604-8610.
